# Cognitive and physical activity and dementia: A 44-year longitudinal population study of women

**DOI:** 10.1212/WNL.0000000000007021

**Published:** 2019-03-19

**Authors:** Jenna Najar, Svante Östling, Pia Gudmundsson, Valter Sundh, Lena Johansson, Silke Kern, Xinxin Guo, Tore Hällström, Ingmar Skoog

**Affiliations:** From the Department of Psychiatry and Neurochemistry (J.N., S.O., P.G., V.S., L.J., S.K., X.G., T.H., I.S.), Institute of Neuroscience and Physiology, The Sahlgrenska Academy at Gothenburg University, Mölndal; and the Department of Epidemiology and Social Medicine (V.S.), The Sahlgrenska Academy at Gothenburg University, Gothenburg, Sweden.

## Abstract

**Objective:**

To investigate whether cognitive and physical activities in midlife are associated with reduced risk of dementia and dementia subtypes in women followed for 44 years.

**Methods:**

A population-based sample of 800 women aged 38–54 years (mean age 47 years) was followed from 1968 to 2012. Cognitive (artistic, intellectual, manual, religious, and club) and physical activity were assessed at baseline. During follow-up, dementia (n = 194), Alzheimer disease (n = 102), vascular dementia (n = 27), mixed dementia (n = 41), and dementia with cerebrovascular disease (n = 81) were diagnosed according to established criteria based on information from neuropsychiatric examinations, informant interviews, hospital records, and registry data. Cox regression models were used with adjustment for age, education, socioeconomic status, hypertension, body mass index, cigarette smoking, diabetes mellitus, angina pectoris, stress, and major depression.

**Results:**

We found that cognitive activity in midlife was associated with a reduced risk of total dementia (hazard ratio [HR] 0.66; 95% confidence interval [CI] 0.49–0.89) and Alzheimer disease (HR 0.54; 95% CI 0.36–0.82) during follow-up. Physical activity in midlife was associated with a reduced risk of mixed dementia (HR 0.43; 95% CI 0.22–0.86) and dementia with cerebrovascular disease (HR 0.47; 95% CI 0.28–0.78). The results were similar after excluding those who developed dementia before 1990 (n = 21), except that physical activity was then also associated with reduced risk of total dementia (HR 0.67; 95% CI 0.46–0.99).

**Conclusion:**

Our findings suggests that midlife cognitive and physical activities are independently associated with reduced risk of dementia and dementia subtypes. The results indicate that these midlife activities may have a role in preserving cognitive health in old age.

Several longitudinal studies report that cognitive^1–4^ and physical activity^3,5,6^ may reduce the risk of dementia. Others have not confirmed these findings.^1,2,4^ Most studies have a high mean age at baseline and a short observation time. Low levels of cognitive and physical activities may thus be a consequence of preclinical dementia in studies with short follow-up.^7^

Among long-term follow-up studies, a prospective co-twin control study on male twin pairs, followed over 20 years, showed that midlife cognitive activity was associated with a 26% risk reduction for dementia. However, midlife physical activity did not modify dementia risk.^8^ The PAQUID study showed that engagement in both physical and cognitive activity reduced risk of dementia over a 20-year follow-up. However, none of the studies investigated the activities in relation to other dementia subtypes than Alzheimer disease (AD).^8,9^

It was recently reported that cardiovascular fitness in midlife was associated with decreased risk of dementia among 191 women followed over 44 years.^10^ Other studies with long observation time reported an association between physical activity and reduced risk of dementia,^11–14^ while a study with a 27-year follow-up did not.^15^ However, the ascertainment of dementia in the latter study was based on electronic health records and did not include dementia subtypes. None of the mentioned studies examined the independent effects of physical and cognitive activity on dementia risk.

We used data from the Prospective Population study of Women with 44 years of follow-up to investigate the role of midlife cognitive and physical activity on risk of late-life dementia disorders. In separate analyses, we excluded those who developed dementia during the first 22 years to minimize the possibility that associations were due to preclinical dementia.

## Methods

As part of the Gothenburg H70 Birth Cohort Studies, we examined incidence of dementia in the Prospective Population Study of Women in Gothenburg, Sweden. In 1968–1969, a systematically selected sample of 899 women (participation rate 89%) aged 38, 46, 50, and 54 years (mean age 47 years) were invited to a health examination that included both physical and psychiatric investigations.^16^ The women were selected from the Swedish Population Registry based on specific birth dates to yield a representative sample.^16^ There were no differences in age, socioeconomic status (SES), work outside the home, or history of contact with mental health services among those who participated at baseline in 1968 (n = 800) compared to nonparticipants (n = 99).^17^

After the first examination at baseline in 1968–1969, participants were re-examined in relation to dementia in 1974–1975 (n = 677), 1980–1981 (n = 629), 1992–1993 (n = 371), 2000–2001 (n = 363), 2005–2006 (n = 299), and 2009–2010 (n = 269). Response rates among survivors were 85% in 1974–1975, 73% in 1980–1981, 67% in 1992–1993, 73% in 2000–2001, 75% in 2005–2006, and 67% in 2009–2010. Dementia diagnoses were also collected from the Swedish Hospital Discharge Registry for all individuals discharged from hospitals on a nationwide basis from 1978 to 2012.

### Standard protocol approvals, registrations, and patient consents

The Ethics Committee for Medical Research at the University of Gothenburg approved the study and all participants gave informed consent to participate according to the Helsinki declaration.

### Cognitive activity

Five cognitive activities were assessed during semi-structured psychiatric interviews: intellectual, artistic, manual, club, and religious. The frequency of each activity was rated as no/low (score 0), moderate (score 1), or high (score 2). None/low was rated when the women did not participate in any of the different activities described above. A moderate degree of intellectual activity included for example reading a book during the last 6 months, while a high degree was exemplified with reading more books or writing. A moderate degree of artistic activity included visiting a concert, theatre, or an art exhibition during the last 6 months, while a high degree of activity included more frequent visits, playing an instrument, singing in a choir, or painting. A moderate degree of manual activity included for example needlework in last 6 months or gardening during the last year, whereas a high degree involved several interests or frequent activities. A moderate degree of club activity included for example having a membership, while a high degree of activity meant having a board membership. A moderate degree of religious activity included church attendance at least a few times during the last year and a high degree included church attendance at least 12 times during the last year.

The cognitive activities were assembled to a sum score based on the frequency level (score 0–2), as described above. The sum score was dichotomized as 0–2 vs 3–10 based on the median of engagement.

### Physical activity

The participants were interviewed regarding their levels of physical activity using the Saltin-Grimby Physical Activity Level Scale. This scale has shown predictive validity in relation to cardiovascular risk factors.^18^ Based on this measure, the women were assigned to 4 groups. Group 1 was completely inactive, for example at most watching television and going to the movies. Group 2 engaged in light physical activity for a minimum of 4 h/wk, such as walking, gardening, bowling, or cycling for half an hour a day. Group 3 had regular physical training, such as running, tennis, or swimming, for at least 2–3 h/wk. Group 4 had regular–intense physical training such as heavy exercise, for example running or swimming several times/week, or engaging in competitive sports.

Physical activity was dichotomized as inactive (group 1) vs active (group 2–4) based on the distribution of engagements.

### Neuropsychiatric examination

Neuropsychiatric examinations were performed by psychiatrists in 1968–1969, 1974–1975, 1980–1981, and 1992–1993, and by experienced psychiatric research nurses in 2000–2003, 2005–2006, and 2009–2010. The examinations were semi-structured and included comprehensive psychiatric examinations and an extensive battery of neuropsychological tests.^19^ Close informant interviews were performed by psychiatric nurses in 1992–1993, 2000–2003, 2005–2006, and 2009–2010. The interviews were semi-structured and comprised questions about changes in behavior and intellectual function, psychiatric symptoms, activities of daily living, and in cases of dementia, age at onset and disease course.^20^

### Diagnosis of dementia

The diagnosis of dementia at each examination was based on the criteria in the DSM-III-R, using information from neuropsychiatric examinations and close informant interviews, as described in detail previously.^20,21^ Dementia diagnoses for individuals lost to follow-up were based on information obtained from the Swedish Hospital Discharge Registry 1978–2012.^19^ These diagnoses had to be compatible with DSM-III-R criteria.

AD was diagnosed according to the criteria of the National Institute of Neurologic and Communicative Disorders and Stroke and the Alzheimer's Disease and Related Disorders Association (NINCDS-ADRDA).^22^ Vascular dementia (VaD) was diagnosed with criteria similar to those outlined by the National Institute of Neurological Disorders and Stroke and the Association Internationale pour la Recherce et l'Enseignement en Neurosciences (NINDS-AIREN), as described previously.^23^ VaD was diagnosed when there was a temporal relationship (within 1 year) between a history of acute focal neurologic symptoms and signs (hemiparesis or motor aphasia) and the first symptoms of dementia. Mixed dementia was diagnosed when both AD and cerebrovascular disease (CVD) were judged to contribute to dementia. We also created a group “dementia with CVD,” which included individuals with dementia and stroke without considering the temporal relationship between the occurrence of dementia and stroke. Practically, this group includes VaD, mixed dementia, and AD with CVD.^19^

Age at onset was determined based on information provided by close informants, the hospital discharge register, and the examinations. If no information could be obtained from these sources, the age at onset was determined as the midpoint between the last examination free from dementia and the first with a dementia diagnosis.

### Potential confounders

Information on potential confounders was obtained at the examination in 1968–1969 (education, SES, hypertension, smoking, diabetes mellitus, angina pectoris, psychological stress, major depression). Education was dichotomized as compulsory (6 years for those born in 1908–1922 and 7 years for those born in 1930) vs more than that. SES was based on husband's occupation for married women and own occupation for unmarried women and was defined as high (upper middle class and above), medium (lower middle class), and low (working class).^24^ Hypertension was defined as systolic blood pressure ≥160 mm Hg or diastolic blood pressure ≥95 mm Hg, or taking antihypertensive medications. Body mass index was calculated as kg/m^2^ and categorized according to international standards. Current cigarette smoking was defined as number of cigarettes per day. Diabetes mellitus was defined as a diagnosis confirmed by a doctor, being on antidiabetes drugs, or having 2 fasting blood glucose values of 7.0 mmol/L or more. Angina pectoris was defined according to the Rose criteria. Psychological stress was defined as frequent symptoms of stress symptoms, such as tension, nervousness, and sleep disturbance (≥1 month), and diverted in 2 groups: no/occasional stress vs frequent/constant stress. Major depressive episode was diagnosed according to the DSM-III-R.^21^

### Statistical analysis

Sociodemographic and health characteristics are presented as numbers, mean and median values, SD, and interquartile range. The association between midlife cognitive and physical activity and incidence of dementia disorders was analyzed by Cox regression models. The associations are presented as hazards ratios and 95% confidence intervals in 3 models. The proportional hazard assumption was met for all Cox regression models.

The first model (model 1) analyzed cognitive and physical activity separately and was adjusted for age. The second model (model 2) included age and both activities simultaneously. The third model (model 3) included age, both activities, and relevant covariates. To select relevant covariates for model 3, a primary analysis was performed, where each potential confounder was analyzed in relation to dementia disorders using age and cognitive and physical activity as confounders. The covariates that were not significantly related to dementia disorders were omitted (using *p* < 0.3 to include covariates that might affect the studied associations, even if these were not formally statistically significantly associated with the factor). The covariates used in model 3 were SES and cigarette smoking for total dementia; major depression and SES for AD; SES and hypertension for VaD; education, smoking cigarettes, and hypertension for mixed dementia; and education, SES, smoking cigarettes, and hypertension for dementia with CVD.

In addition, to investigate a potential dose–response relationship among the significant associations between cognitive and physical activity and dementia disorders, sensitivity analyses were performed using Cox regression models. These analyses included dementia disorders, age, and relevant covariates (as described above). Cognitive activity was divided into 4 quartiles (both in contrast tests with first quartile as reference, and test of linear associations by quartiles) and physical activity divided into 3 tertiles (both in contrast tests with the first tertile as reference, and in tests of linear associations by tertiles).

Finally, sensitivity analyses were performed with the exclusion of those who developed dementia before 1990 (22 years after baseline) to minimize the possibility of reverse causation, i.e., that findings would be caused by preclinical dementia. The cutoff is based on previously published studies that report a possible occurrence of preclinical dementia 20–30 years before dementia onset.^7,15^

Time at risk for women who did not develop dementia was calculated from the date of the baseline examination to (1) the date of the last follow-up examination for participants in 2009; (2) the date of death for those who died during follow-up; or (3) December 31, 2012, for surviving drop-outs. For women who developed dementia, time at risk was calculated from the date of the baseline examination to the estimated year of dementia onset.

### Data availability statement

Any data not published within the article are available from the corresponding author on reasonable request.

## Results

Sociodemographic factors, health characteristics, and levels of leisure cognitive and physical activity at baseline in 1968 are presented in table 1. The sum score of cognitive activities demonstrated a Poisson distribution with the peak of activity at sum score 2 (21.0%) and 3 (19.4%). The distribution of physical activity over the 4 groups shows that the majority of the women (70.0%) were physically active on a regular basis (group 2), and a minority (12.0%) had regular–intense physical training (group 3 and 4).

**Table 1:**
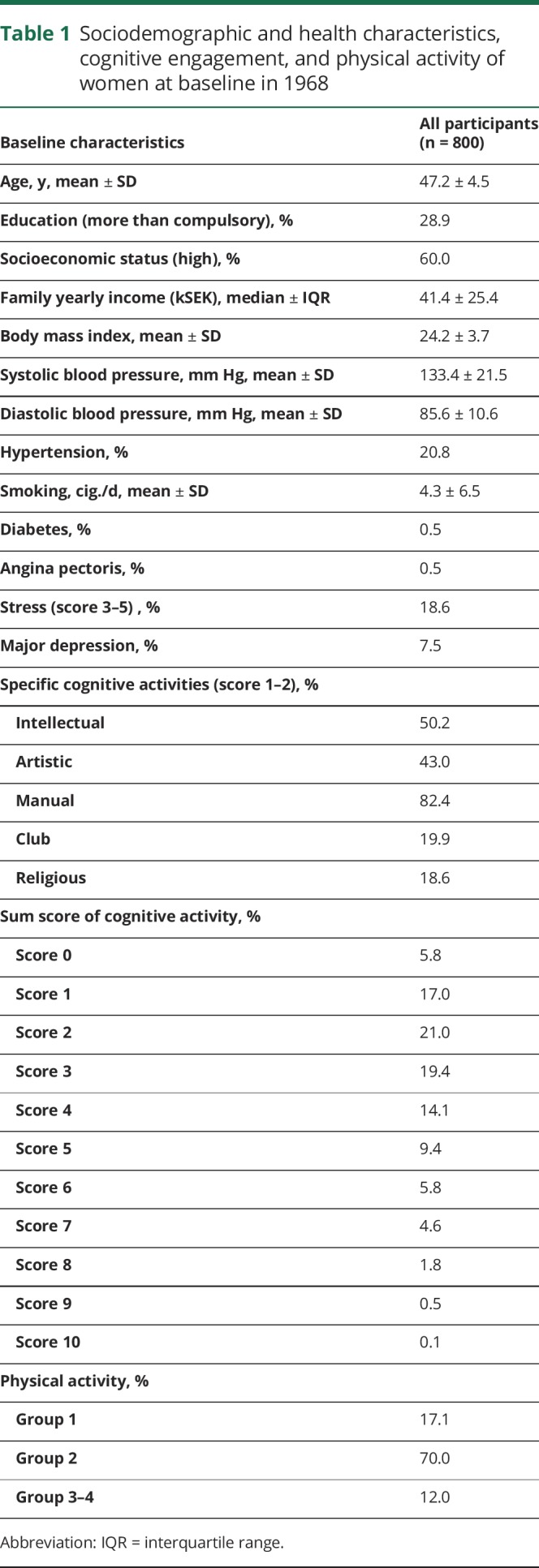
Sociodemographic and health characteristics, cognitive engagement, and physical activity of women at baseline in 1968

From 1968 to 2012 (mean follow-up 44 years, SD 9.8), 194 (24.3%) women developed dementia during 26,322 person-years of follow-up, including 102 (52.6%) with AD, 27 (13.9%) with VaD, 41 (21.1%) with mixed dementia, and 14 (7.2%) with other dementias. In total, 81 (41.8%) of those who developed dementia had dementia with CVD. The mean time from the baseline examination in 1968 to dementia onset was 31.5 years (SD 7.7) (21 had dementia onset before 1990). Mean age at dementia onset was 79.8 years (SD 7.7). The number of participants who died during follow-up was 596 (74.5%). Mean age at death was 80.0 years (SD 9.5).

As can be seen in table 2, midlife cognitive activities were associated with a reduced risk of total dementia and AD in all 3 models.

**Table 2:**
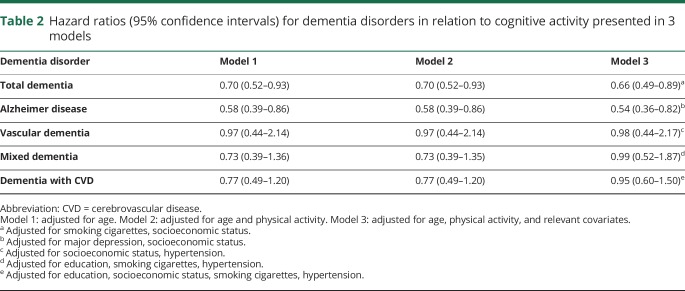
Hazard ratios (95% confidence intervals) for dementia disorders in relation to cognitive activity presented in 3 models

Table 3 shows that midlife physical activity was associated with a reduced risk of mixed dementia and dementia with CVD in all 3 models.

**Table 3:**
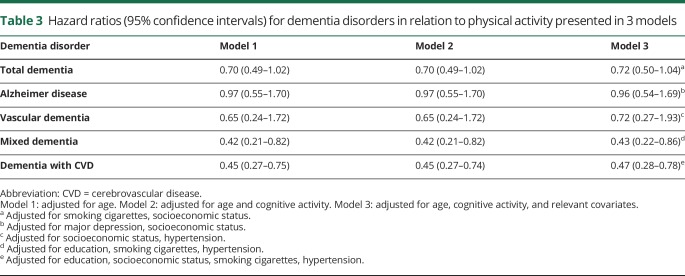
Hazard ratios (95% confidence intervals) for dementia disorders in relation to physical activity presented in 3 models

Further analyses were conducted to investigate possible linear associations (table 4). A decreased risk of total dementia and AD was found for the third and fourth quartile of cognitive activity. A similar linear association was found for physical activity and mixed dementia. However, no such association was found between physical activity and dementia with CVD.

**Table 4:**
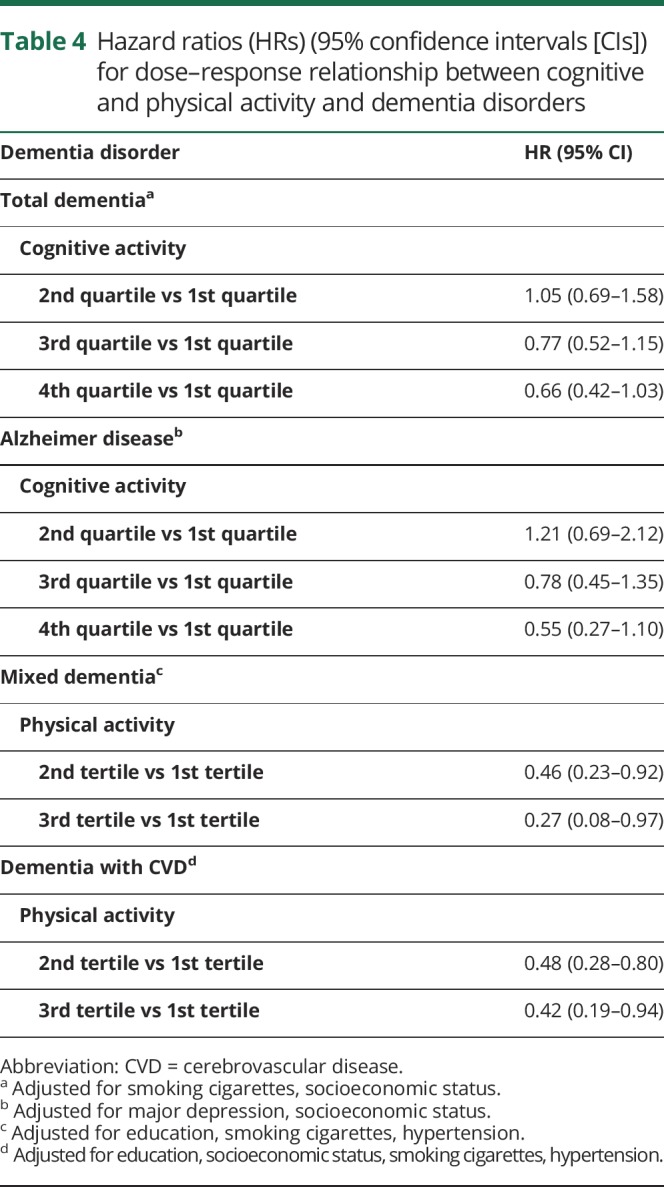
Hazard ratios (HRs) (95% confidence intervals [CIs]) for dose–response relationship between cognitive and physical activity and dementia disorders

Finally, sensitivity analyses with exclusion of those who developed dementia before 1990 (n = 21) showed similar results, except that in these analyses physical activity was also associated with reduced risk of total dementia in all 3 models (table 5). Included in the analysis were 174 women who developed dementia during the 26,014 person-years of follow-up, and 93 women were diagnosed with AD, 25 with VaD, 34 with mixed dementia, and 72 with dementia with CVD.

**Table 5:**
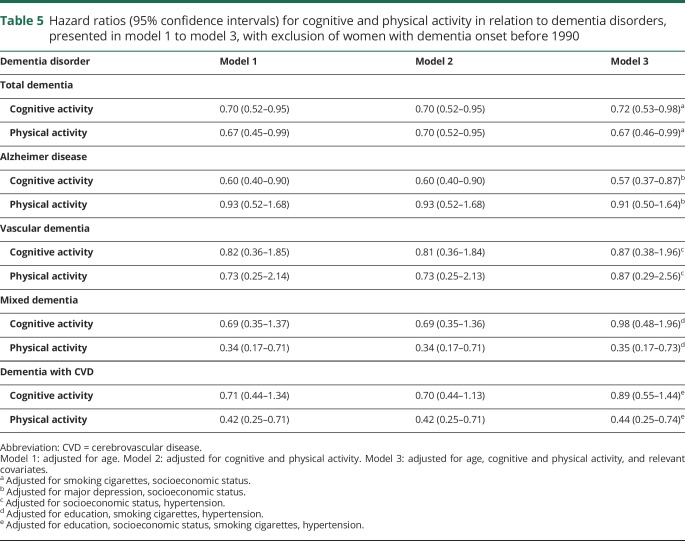
Hazard ratios (95% confidence intervals) for cognitive and physical activity in relation to dementia disorders, presented in model 1 to model 3, with exclusion of women with dementia onset before 1990

## Discussion

We found that higher cognitive and physical activity in midlife were independently related to reduced risk of dementia disorders in a population sample of women followed over 44 years. Cognitive activity in midlife reduced the risk of AD, while physical activity reduced the risk of mixed dementia and dementia with CVD. None of the activities was related to the incidence of VaD.

Our finding that midlife cognitive activities were related to reduced incidence of dementia is similar to several studies conducted among older persons with shorter follow-ups (3–7 years)^1–4^ and 2 studies with longer follow-up (20–40 years).^8,9^ Regarding physical activity, results from previous prospective studies are inconsistent. Three studies with shorter follow-ups (3–7 years) conducted among older people reported that physical activity reduced the risk of AD,^3,5,6^ while 3 studies did not find a relationship.^1,2,4^ Studies on physical activity with long follow-ups (more than 15 years) reported that physical activity was associated with reduced risk of dementia and AD,^11–14^ with the exception of 2 recent studies.^8,15^ Reasons for discrepancies could be that one study was a twin study with rigorous control for genetics and early life exposures,^8^ and the other used electronic health records for the diagnosis of dementia, which might miss true cases of dementia.^15^ In relation to this, the CAIDE study^14^ found an association between midlife physical activity and dementia, when dementia diagnoses were based on clinical examinations, but no association when diagnoses were based on electronic health records.^14^ None of the long-term studies reported on whether physical and cognitive activities were independently related to dementia. In contrast, we found no association between physical activity and AD, but physical activity did reduce risk of mixed dementia and dementia with CVD. Our finding that midlife physical activity reduces risk of dementia in late life is also consistent with the findings that better lung function in midlife and midlife cardiovascular fitness are related to reduced risk of dementia.^10,19^

A possible explanation for our findings may be that midlife cognitive activity increases cognitive reserve. Two kinds of reserve are suggested to compensate for brain damage: brain reserve and cognitive reserve.^25^ Brain reserve is a quantitative model, such as brain size and neuronal count, while cognitive reserve refers to how flexible and efficiently one can make use of available brain reserve.^25^ According to the cognitive reserve model, individuals with higher cognitive reserve require greater decrease in cortical thickness, lower levels of amyloid peptides in CSF, and greater regional atrophy before clinical symptom appears.^25^ In support of this, the Nun Study reported that intelligence early in life, measured as linguistic ability, was associated with reduced risk of dementia half a century later.^26^ An autopsy study reported that greater early–midlife participation in cognitive activities might prevent or slow the deposition of β-amyloid and thus may delay the onset and progression of AD, while there was no association with physical activity.^27^ The association between physical activity and cardiovascular risk factors and stroke may explain the benefits from physical activity on the reduced incidence of mixed dementia and dementia with CVD. Our results showed no relationship between physical activity and VaD. However, the associations moved in the same direction. One reason for this result may be the low statistical power, as we had few cases of VaD.

Despite our long period of follow-up, it cannot be completely ruled out that low cognitive and physical activities are manifestations of very early pathologic processes in dementia disorders. Our measures of cognitive and physical activities were made 24–40 years before onset of dementia, and are thus on the borderline of the first possible pathologic processes of dementia. To elucidate this issue, a sensitivity analysis was conducted with the exclusion of those who developed dementia before 1990 (22 years after baseline). The associations remained unchanged, except that the association between physical activity and dementia became stronger.

Among the strengths of the study are the representative, systematically selected, and well-characterized population sample of women, the long observation period, and the low baseline age among the participants. In addition, psychiatrists or psychiatric nurses performed psychiatric examinations and multiple sources of information were used to detect and diagnose dementia.

Limitations of the study also need to be addressed. First, cumulative attrition is a problem in long-term follow-up studies. Although this issue was partly alleviated by using medical records and the hospital registry to diagnose dementia in those lost to follow-up, these sources underestimate the number of dementia cases. It should be noted that almost all people in Sweden receive their hospital treatment within the public health care system and that the Swedish Hospital Discharge Register covers the entire country. Furthermore, the incidence of dementia in this study is similar to what has been reported in previous studies.^28^ Second, in a long period of follow-up as in our study, the competing risk of death may have affected the results. The use of risk time in the Cox regressions partly accounts for this. Third, we assessed cognitive and physical activity only at baseline. However, midlife cognitive and physical activities tend to continue into old age,^29^ and may thus indirectly reflect late-life activities. Moreover, cognitive benefits obtained from cognitive activities, once established, may remain relatively stable with age.^29^ Fourth, the reliability of the assessment of cognitive activity is not known. However, the measure could be regarded as having satisfactory long-term predictive validity, as the results agree with most other similar studies, and because cognitive activity was specifically related to AD but not to other dementia subtypes. Fifth, all members of the sample are women, Caucasian, and living in Sweden, thereby limiting the possibility to generalize to other populations, and to men. Sixth, we used a rather crude assessment for educational level. However, the variance in education was small, thus more than 70% of the population only had 6–7 years of compulsory education. Seventh, we cannot exclude the possibility that persons with high cognitive reserve engage more in cognitive activities than other persons. However, to account for this, we controlled for education in the analyses.

We found that higher levels of cognitive and physical activity in midlife were independently associated with reduced risk of dementia and dementia subtypes in late life. Cognitive activity primarily reduced incidence of AD, while physical activity reduced the incidence of mixed dementia and dementia with CVD. The results indicate that activities in midlife might play a role in the prevention of late-life dementia.

## Glossary

AD: Alzheimer disease
CVD: cerebrovascular disease
DSM-III-R: *Diagnostic and Statistical Manual of Mental Disorders, 3rd edition, revised*
SES: socioeconomic status
VaD: vascular dementia
